# UPR^mt^ and coordinated UPR^ER^ in type 2 diabetes

**DOI:** 10.3389/fcell.2022.974083

**Published:** 2022-09-16

**Authors:** Zhanfang Kang, Feng Chen, Wanhui Wu, Rui Liu, Tianda Chen, Fang Xu

**Affiliations:** ^1^ Department of Basic Medical Research, Qingyuan People’s Hospital, The Sixth Affiliated Hospital of Guangzhou Medical University, Qingyuan, China; ^2^ Guangzhou Municipal and Guangdong Provincial Key Laboratory of Protein Modification and Degradation, School of Basic Medical Sciences, Guangzhou Medical University, Guangzhou, China

**Keywords:** UPR^mt^, UPR, unfolded protein response, T2D, PERK (PKR-like endoplasmic reticulum kinase), mitochondia

## Abstract

The mitochondrial unfolded protein response (UPR^mt^) is a molecular mechanism that maintains mitochondrial proteostasis under stress and is closely related to various metabolic diseases, such as type 2 diabetes (T2D). Similarly, the unfolded protein response of the endoplasmic reticulum (UPR^ER^) is responsible for maintaining proteomic stability in the endoplasmic reticulum (ER). Since the mitochondria and endoplasmic reticulum are the primary centers of energy metabolism and protein synthesis in cells, respectively, a synergistic mechanism must exist between UPR^mt^ and UPR^ER^ to cooperatively resist stresses such as hyperglycemia in T2D. Increasing evidence suggests that the protein kinase RNA (PKR)-like endoplasmic reticulum kinase (PERK) signaling pathway is likely an important node for coordinating UPR^mt^ and UPR^ER^. The PERK pathway is activated in both UPR^mt^ and UPR^ER^, and its downstream molecules perform important functions. In this review, we discuss the mechanisms of UPR^mt^, UPR^ER^ and their crosstalk in T2D.

## Introduction

There are many internal and external challenges for cells that living systems must manage to sustain normal and healthy living activities. Therefore, some organelle-specific stress responses have evolved to deal with these external factors, such as the unfolded protein response in endoplasmic reticulum and mitochondria (UPR^ER^ and UPR^mt^, respectively) (Wang and Kaufman, 2012; Jovaisaite et al., 2014; Lee and Ozcan, 2014). The endoplasmic reticulum (ER) is a compartment that facilitates the synthesis, folding, modification and transportation of proteins. Therefore, the proteostasis in ER is so critical that cells have evolved relevant stress response signals to manage the homeostasis disruption. For example, UPR^ER^ is a regulation mechanism that disposes of the proteostasis imbalance in the ER (Kaufman, 2002; Ron, 2002; Rutkowski and Kaufman, 2004; Ron and Walter, 2007). Furthermore, the mitochondrion can code proteins related to oxidative phosphorylation (OXPHOS), which has an intimate connection with energy metabolism. When mitochondria malfunction and there is no remedial measure, cells die. Hence, a remedial measure or surveillance program is important for maintaining living systems (Wouters and Koritzinsky, 2008; Pellegrino et al., 2013; Nagelkerke et al., 2014; Münch and Harper, 2016; Qureshi et al., 2017). One such a measure, UPR^mt^, consistently monitors the environment in mitochondria, which is vital to the organelle’s normal functioning (Yoneda et al., 2004; Benedetti et al., 2006; Houtkooper et al., 2013). Recent evidence suggests that if cells cannot dispose of various stresses and resolve the disruption of homeostasis rapidly, multiple disorders such as metabolic and neurodegenerative diseases occur (Wang and Kaufman, 2012; Jovaisaite et al., 2014; Lee and Ozcan, 2014). In addition, the cell is a highly dynamic but orderly system wherein the organelles coordinate to ensure its healthy and orderly activities (Csordás et al., 2006; West et al., 2011; Grimm, 2012; Allison et al., 2017). Mitochondria ER contacts (MERCs), also called mitochondria associated membranes (MAMs) are composed of ER and mitochondrion membranes, house many molecules that are situated at contact sites, and facilitate signal exchange between two compartments (Vance, 2014; Ilacqua et al., 2017; Zhang et al., 2021a; Sukhorukov et al., 2022). Therefore, organelle-specific stress responses, such as UPR^ER^ and UPR^mt^, can also coordinate to manage stresses so as to maintain cellular homeostasis and prevent the development of multiple disorders. In this study, we evaluate UPR^mt^ and UPR^ER^, as well as the crosstalk between them, and highlight the coordinative impact of them in T2D.

### Characterization of the UPR^mt^

Mitochondria are organelles with double membranes, which contain proteins and RNAs encoded by the nuclear genome and are also capable of encoding proteins and RNAs related to OXPHOS (Sloan et al., 2018). Mitochondria that do not work normally cannot provide ATP for the efficient functioning of cells with their highly dynamic and interconnected mitochondrial network (Wilkins et al., 2017; Sloan et al., 2018; Zhang et al., 2018). In addition to producing energy, mitochondria also play an indispensable role in other signaling cascades, such as the signaling axis that regulates apoptosis (Wilkins et al., 2017; Zhang et al., 2018; Dall and Færgeman, 2019). Simultaneously, mitochondria have waste removal and utilization systems (Vakifahmetoglu-Norberg et al., 2017). Therefore, mitochondrial proteostasis is indispensable, and without it, mitochondria cannot maintain their homeostasis and perform their normal tasks (Roger et al., 2017). The bulk of proteins in mitochondria are encoded by nuclear genes, which are then translated through free ribosomes (not through co-translation), and can finally be transported to the mitochondria to produce their marked effect. However, mitochondrial DNA that codes for 13 OXPHOS reaction center proteins are translated into the mitochondrial matrix (Hansen and Herrmann, 2019). Proteins encoded by nuclear genes or proteins encoded by mitochondrial DNA must be correctly folded and subjected to quality control to sustain mitochondrial proteostasis. Misfolded proteins must be refolded or degraded to rebuild mitochondrial proteostasis (Schmidt et al., 2010; Ott et al., 2016).

The UPR^mt^ is a mitochondrial stress response that maintains proteostasis in the mitochondria (Haynes and Ron, 2010; Schmidt et al., 2010; Durieux et al., 2011; Ott et al., 2016; Xie et al., 2017; Smyrnias et al., 2019). When proteins that need to be processed exceed the protein-handling capacity of the mitochondria, UPR^mt^ initiates an increase in protein degradation and expansion of the mitochondrial matrix folding capacity to restore protein homeostasis (Ryan and Hoogenraad, 2007). Restoring protein homeostasis in mitochondria relies on chaperones that increase protein folding and block protein aggregation and are imperative to all cells (Bukau et al., 2006). Different stressors, such as oxidative stress, infections, and alterations in metabolism, are not beneficial for protein folding (Aldridge et al., 2007; Ron and Walter, 2007). In addition, because the influx of mitochondrial precursor proteins and the correct assembly of nuclear and mitochondrial DNA-encoded proteins are essential for the healthy survival of cells, restoration of mitochondrial protein homeostasis encounters unique challenges (Ryan and Hoogenraad, 2007). A series of molecular chaperones and proteases are indispensable for protein proteostasis in mitochondria, participating in the transduction pathway of UPR^mt^ as mediators and targets (Zhao et al., 2002; Aldridge et al., 2007).

In addition to proteostasis disturbances, the disruption of mitochondrial metabolic processes also activates UPR^mt^. Multiple drugs suppress OXPHOS and activate UPR^mt^ (Yoneda et al., 2004; Nargund et al., 2012; Rossmanith et al., 2012; Houtkooper et al., 2013; Zhang et al., 2021b), including inhibitors of complex I, such as rotenone and petasin (Heishima et al., 2021), an inhibitor of complex III, antimycin, and inhibitors of complex V, oligomycin and paraquat. Fumarate hydratase, an enzyme that converts fumarate to malate during the tricarboxylic acid cycle (TCA), can be inhibited to activate UPR^mt^, demonstrating that the mitochondrial stress response can be activated not only by mitochondrial proteostasis disruption but also by metabolic disturbances (Wang et al., 2016).

UPR^mt^ has been associated with multiple biological processes, including development (Sukhorukov et al., 2022), innate immune signaling (Zhu et al., 2021), aging (Mohrin et al., 2015; Lin et al., 2016; Wang et al., 2016; Zhu et al., 2021) and cardioprotection (Qureshi et al., 2017). Once the UPR^mt^ signal is prolonged to a chronic state potentially maladaptive to organelles, it presages a therapeutic target for a broad spectrum of illnesses. Rapid activation of UPR^mt^ facilitates the adaptability of cells to environmental stresses and physiological stimuli. The transduction signal of UPR^mt^ promotes the proliferation of defective mitochondrial genomes, which ultimately leads to congenital metabolic abnormalities (Haynes et al., 2007; Yan et al., 2020). In addition, dysregulated UPR^mt^ signaling leads to the development of diseases such as cancer (Martinus et al., 1996; Shpilka et al., 2021), neurodegenerative disorders (Roger et al., 2017; Hansen and Herrmann, 2019), fatty liver, and diabetes (Haynes et al., 2007; Yan et al., 2020). Also, UPR^mt^ promotes host tolerance and protects against pathogenic infections (Mehrbod et al., 2019).

Although UPR^mt^ was initially discovered and characterized in mammalian cells (Martinus et al., 1996; Zhao et al., 2002), research on UPR^mt^ has been more thorough in *Caenorhabditis elegans* (*C. elegans*). In *C. elegans*, mitochondrial matrix proteins can be hydrolyzed to generate short peptides by CLPP-1 when the processing capability of chaperone proteins cannot afford the aggregation of misfolded and unassembled proteins (Nicolas et al., 2019). HAF-1, a mitochondria-localized ATP-binding cassette protein, causes an influx of these peptides in the mitochondrial matrix into the cytoplasm in an ATP-dependent manner, thereby inhibiting the import of mitochondrial proteins (Haynes et al., 2010). In addition, mitochondrial stressors diminish mitochondrial membrane potential, which hinders the import of mitochondrial proteins (Rolland et al., 2019). The basic leucine zipper (bZIP) transcription factor ATFS-1, which has two special sequences, the mitochondrial targeting sequence (MTS) and the nuclear localization sequence (NLS), plays a crucial role in the regulation of the UPR^mt^ (Haynes et al., 2010; Nargund et al., 2012). The mitochondrial-to-nuclear communication is mediated by dual subcellular localization sequences (Lin et al., 2016; Sorrentino et al., 2017). Thus, the import efficiency of mitochondrial proteins is a possible indicator of the general mitochondrial function in cells where ATFS-1 is used as a sensor. Under normal circumstances, ATFS-1 is transported into the mitochondrial matrix, where the protein is degraded. However, in times of stress, the influx of ATFS-1 is inhibited, leading to its accumulation in the nucleus; this activates the transcription of a set of genes related to encoding chaperones and proteases participating in UPR^mt^, suggesting that mitochondrial import efficiency is a key regulator of UPR^mt^ activation (Nargund et al., 2012; ; Nargund et al., 2015). DVE-1, a UPR^mt^ regulator capable of binding to the promoter of heat shock protein (HSP60), has recently been found downstream of mitochondrial dysfunction-driven chromatin reorganization. Histone methyltransferases, MET-2 and LIN-65, are involved in chromatin reorganization, and regardless of which is inhibited, they would reduce the methylation of histone H3K9 and block the induction of UPR^mt^ downstream of CLPP-1. In addition, it was found that MET-2 was necessary for the accumulation of LIN-65 in the nucleus, which could promote the opening of a portion of chromatin, facilitating the binding to open regions and the transcriptional activity of DVE-1 (Tian et al., 2016; Wang et al., 2019). Furthermore, demethylation of JMJD-1.2 and JMJD-3.1 in histone H3K27 is also required for UPR^mt^ induction. Importantly, overexpression of any of these demethylases leads to a transcriptional response resembling that observed during OXPHOS dysfunction (Merkwirth et al., 2016). These studies highlight that the DVE-1-mediated transcriptional response in times of mitochondrial stress is an important precursor for chromatin remodeling. Both ATFS-1 and DVE-1 are regulated by small ubiquitin-like modifier (SUMO) proteins, and the SUMO moieties of the peptidase have been previously identified to be required for ULP-4 cleavage to UPR^mt^ induction (Liu et al., 2014a). AVE-1 and ATFS-1 were SUMOylated at K327 and K326, respectively, leading to accelerated proteolysis of ATFS-1 and import of DVE-1. In the absence of ulp-4, mutations that eliminate SUMOylation of these transcription factors also activate UPR^mt^ (Gao et al., 2019).

Although there is considerable overlap in the regulatory mechanisms between mammalian UPR^mt^ and *C. elegans* UPR^mt^, mammalian UPR^mt^ regulatory mechanisms are more sophisticated (Fiorese et al., 2016; Münch and Harper, 2016; Quirós et al., 2017). In mammalian cells facing mitochondrial stress, eIF2α-specific kinases such as GCN2, PERK, PKR, and HRI are activated for the phosphorylation of the translation initiation factor eIF2α, which restrains the synthesis of general proteins while increasing the translation of some specific proteins (Barbosa et al., 2013; Pakos-Zebrucka et al., 2016). For example, CHOP, ATF4, and ATF5 bZIP transcription factors were increased in UPR^mt^. ATF5, a functional mammalian homolog of the transcription factor ATFS-1, which has been shown in *C. elegans* to regulate UPR^mt^, elicits a transcriptional response (; Persengiev et al., 2002). Like ATFS-1 in *C. elegans*, ATF5 harbors a sequence similar to MTS, which allows it to be degraded through the mitochondrial membrane. However, during UPR^mt^, it could not efficiently import into the mitochondria to accumulate in the nucleus and induce the expression of genes that have a bearing on several mitochondrial chaperones and proteases, a process that promotes OXPHOS and cell growth during mitochondrial homeostasis disorder to help cells adapt and survive (Silva et al., 2009; Tyynismaa et al., 2010; Martínez-Reyes et al., 2012; Michel et al., 2015; Münch and Harper, 2016; Quirós et al., 2017). In addition to ATF5, two other bZIP transcription factors, ATF4 and CHOP, also participate in UPR^mt^ activation, and an intimate connection exists between ATF4, CHOP, and ATF5 during times of mitochondrial dysfunction. Both CHOP and ATF4 regulate mitochondrial proteins by inducing the transcription of ATF5 (Hatano et al., 2013; Zhu et al., 2021). Furthermore, the transcription factor c-Jun is activated by c-Jun N-terminal kinase (JNK)2 and dsRNA-activated protein kinase (PKR) during stress, which subsequently attaches to AP-1, triggering the translation of CHOP and C/EBP. CHOP combines with C/EBP to form a dimer that is bound to the promoters of UPR^mt^ genes, which encode chaperones HSP60, CLPP-1, and mitochondrial import components (Jovaisaite et al., 2014; Münch and Harper, 2016). In addition, the kinases of eIF2, including GCN2 and PERK, promote the phosphorylation of elF2, thereby suppressing the synthesis of proteins while facilitating the output of CHOP, ATF4, and ATF5 (Teske et al., 2013; Pakos-Zebrucka et al., 2016). Additionally, heat shock transcription factor 1(HSF1) combines with single-stranded DNA binding protein 1(SSBP1) to generate a complex that increases the yield of heat shock proteins HSP60, HSP10, and mtHSP70 during times of stress (Tan et al., 2015). Members of the sirtuin family, including SIRT3 and SIRT7, may also contribute to the regulation of UPR^mt^ (Papa and Germain, 2014). The buildup of proteins in the intermembrane space (IMS) of the mitochondria generates excessive reactive oxygen species (ROS). In turn, AKT and protein kinase B can be stimulated by excessive ROS, activating the phosphorylated estrogen receptor so that it enhances the activities of proteasome 26S and stimulates the translation of intermembrane space proteases HTRA2, OMI, and the mitochondrial regulator NRF1, hence preserving quality control within the intermembrane space protein (Papa and Germain, 2011).

Moreover, the substrate EPS-8/EPS8 of the epidermal growth factor receptor pathway, as a signaling protein adaptor, plays a role in general homeostasis in mitochondria and the regulation of UPR^mt^ by reorganizing the actin cytoskeleton mediated by integrin (Kaspar et al., 2021; Moehle et al., 2021). Rox1 binds mtDNA and performs a TFAM-like function. By inducing UPR^mt^, Rox1 serves as the initial line of defense (Poveda-Huertes et al., 2020).

### The UPR^ER^

The UPR^ER^ is a set of evolutionarily conserved signaling axes that continuously monitor the quality of proteins that have intimate connections with the protein-folding capacity within the ER lumen (Cox et al., 1993; Cox and Walter, 1996; Sidrauski and Walter, 1997; Yoshida et al., 1998; Niwa et al., 1999; Harding et al., 2000; Tirasophon et al., 2000; Yoshida et al., 2001). When the ER protein-folding capacity is so limited that it cannot satisfy the demand for ER function owing to ER stress, UPR^ER^ is activated, assisting the cell in modifying the organelle’s folding function to restore equilibrium. For example, it can expand the size of the ER lumen and increase the expression of genes encoding proteins related to ER folding capacity. Complementary UPR^ER^ actions coordinate protein homeostasis within the ER lumen. If proteostasis cannot be restored, UPR^ER^ initiates cell-apoptosis signaling pathways that destroy defective cells in an effort to benefit the organism (Lin et al., 2007; Lu et al., 2014a). In mammalian species, three distinct transmembrane UPR^ER^ sensors of unfolded proteins accumulate in tandem to resolve homeostatic disruption (Cox et al., 1993; Cox and Walter, 1996; Sidrauski and Walter, 1997; Yoshida et al., 1998; Niwa et al., 1999; Harding et al., 2000; Tirasophon et al., 2000; Yoshida et al., 2001). These three transmembrane proteins composed of sensors are PERK, membrane-tethered activating transcription factor 6 (ATF6), and inositol-requiring enzyme 1α (IRE1α) (Cox et al., 1993; Cox and Walter, 1996; Sidrauski and Walter, 1997; Yoshida et al., 1998; Yoshida et al., 2001; Calfon et al., 2002; Aragón et al., 2009; Korennykh et al., 2009; Li et al., 2010). In times of ER stress, IRE1, as the most evolutionarily conserved arm of UPR^ER^, oligomerizes in the plane of the ER membrane, which promotes the dissociation of Bip from the luminal domain of IRE1, causing subsequent autophosphorylation and allosteric activation of its cytosolic RNase domain, initiating the non-canonical splicing of the 26-nt intron from X-box binding protein 1 (XBP1) as mRNA, producing XBP1s, which is an active transcription factor. Once XBP1s are encoded and translated, they can enter the nucleus to facilitate the production of chaperones, components of ER-associated degradation (ERAD), and foldases, which participate in protein trafficking, degradation, and folding by driving the activation of UPR^ER^-related genes (Bertolotti et al., 2001; Calfon et al., 2002; Lee et al., 2003; Hollien and Weissman, 2006; Acosta-Alvear et al., 2007; Aragón et al., 2009; Hollien et al., 2009; Korennykh et al., 2009; Silva et al., 2009; Li et al., 2010; Hatano et al., 2013; Michel et al., 2015; Sundaram et al., 2017). In addition to XBP1s activation, active IRE1 can also be degraded. ER-localized mRNA transcripts are regulated by IRE1-dependent decay (RIDD) to limit the influx of client proteins into the ER, thereby reducing the protein-folding load in the organelle (Hollien and Weissman, 2006; Acosta-Alvear et al., 2007; Hollien et al., 2009).

PERK, an ER-resident transmembrane serine/threonine kinase, mediates the second branch of UPR^ER^ (Acosta-Alvear et al., 2007). Similar to IRE1, PERK dissociates with Bip to unlock the dormant status and then homomultimerizes as a transmembrane protein of the ER upon sensing ER stress, leading to its autophosphorylation and activation (Harding et al., 1999; Bertolotti et al., 2000; Harding et al., 2002; Marciniak et al., 2006; Hollien et al., 2009). Activation of PERK phosphorylates eIF2, a critical regulator of protein synthesis (Shi et al., 1998; Harding et al., 1999; Harding et al., 2002). Phosphorylation of eIF2 suppresses general translation, cutting down the expression of proteins into the endoplasmic reticulum, thereby mitigating ER stress in a manner analogous to the attenuation of the protein-folding burden offered by RIDD. Paradoxically, eIF2 phosphorylation promotes preferential translation of a select subset of mRNAs with short open reading frames (uORFs) in their upstream untranslated regions. One of these mRNAs transcripts is ATF4, which, like XBP1s, facilitates the expression of various genes that have something to do with combatting ER dysfunction by improving the cell’s capacity to process native and functional bioproducts, such as the redox homeostasis regulators and importers encoding amino acid (Harding et al., 2003). Recent studies have indicated that PERK has an intimate connection with the formation of ER whorls and is categorized into ER whorls in times of ER stress, and the autophosphorylation activating response of PERK as a result of ER stress and the inhibition of translation cannot be maintained for a long time without the formation of ER whorl precursors. Furthermore, most translocons are isolated into ER whorls during prolonged ER stress, taking translocons away from ribosomes, which augments the interruption of translation. ER whorls are a new kind of ER stress response that attenuates ER perturbations by promoting PERK activation and modulating the co-translation and influx of nascent proteins into the ER (Xu et al., 2021).

**FIGURE 1:**
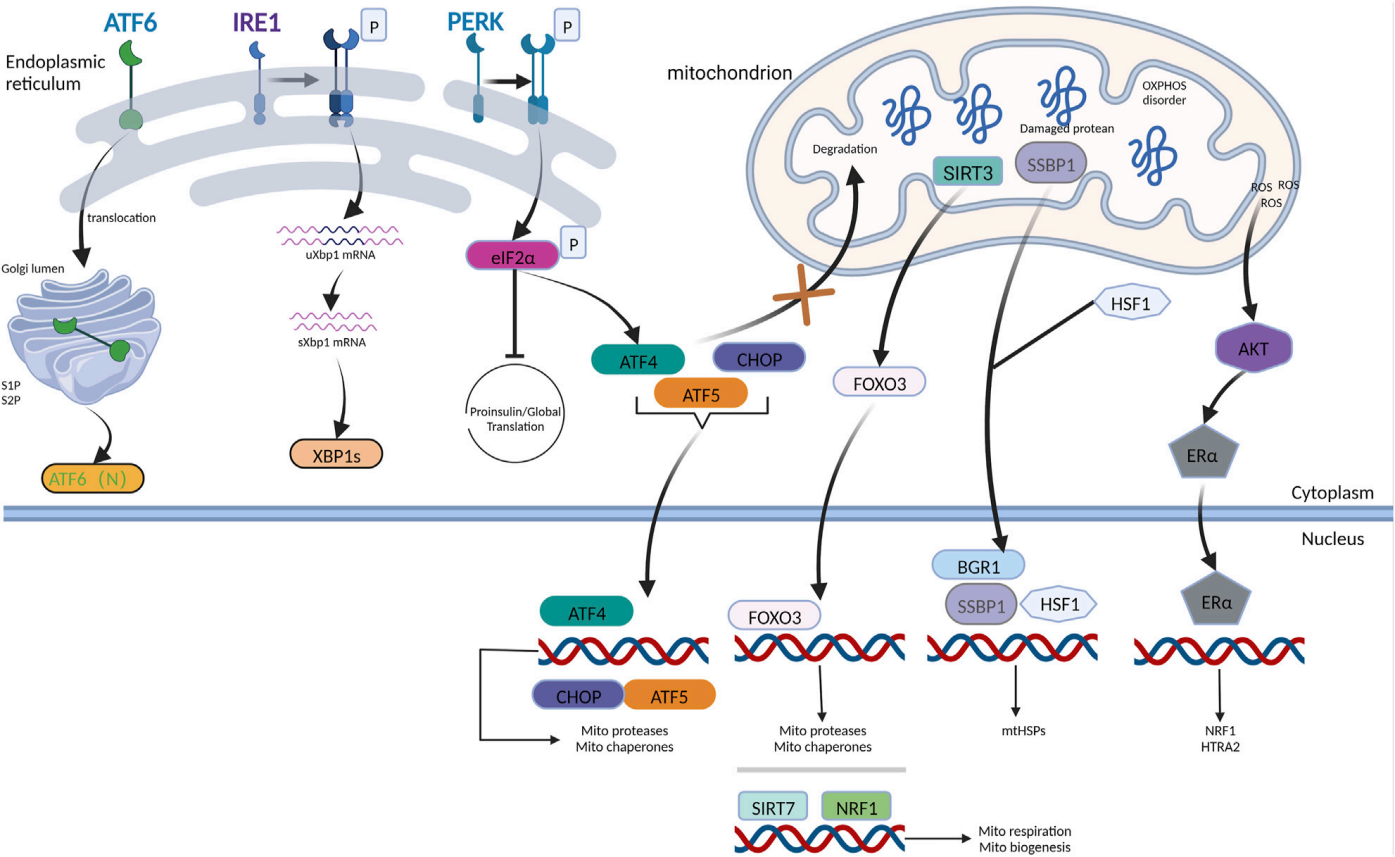
UPR^mt^ mechanism in mammals. The AKT-ER axis is induced by the accumulation of ROS in IMS to trigger the transcription of NRF1 and HTRA2 upon responding to IMS damage. When ATF5 receives the signal derived from the dysfunctional mitochondria, it influxes into the nucleus, accompanied by ATF4 and CHOP, facilitating the production of chaperones and proteases belonging to mitochondria. HSF1 combines with SSBP1, forming a complex bound to the chromatin remodeling protein BRG1, prompting the expression of mtHSPs. Mitophagy and oxidative stress can trigger the transduction of SIRT3-FOXO3 signaling pathways. NRF1 coordinating with SIRT7 reduces the burden of damaged proteins via inhibition of mitochondrial respiration and synthesis. In conclusion, the mammalian UPR^mt^ facilitates mitochondrial recovery and maintains proteostasis via multiple mechanisms. IRE1, PERK, and ATF6 are three ER stress sensors that monitor protein-folding conditions in the ER lumen. PERK-ATF4 axis is the main hub coordinating UPR^ER^ and UPR^mt^.

ATF6 regulates the third branch of the UPR (Figure 1). When the site of two Golgi-localization signals is unmasked via the dissociation of Bip during ER stress, ATF6 is exported to the Golgi apparatus with an accumulation of unfolded proteins, where the entire protein is sequentially and successively digested by site-1 protease (S1P) and site 2 protease (S2P) upon reaching the Golgi apparatus by removing their lumenal domain and transmembrane anchor, respectively, releasing the fragment facing cytosol that is translocated to the nucleus to initiate transcription related to degradation of abnormal proteins (Haze et al., 1999; Wang et al., 2000; Ye et al., 2000). However, ATF6 differs from PERK and IRE1 in that it does not inhibit the influx of client proteins into the ER. Rather, it boosts the ER’s protein processing and degradation capacity, as well as its volume (Wang et al., 2000; Nadanaka et al., 2006; Adachi et al., 2008; Bommiasamy et al., 2009). Therefore, it is suspected that the parallel activation and action of IRE1, PERK, and ATF6 combine to form an adaptive UPR^ER^ phase so that organelles can rapidly respond to ER stress and take timely measures simultaneously to maintain ER-folding equilibrium.

### Cross talk of UPR^mt^ and UPR^ER^

Several studies have demonstrated that UPR^mt^ and UPR^ER^ have intersecting crossroads upon facing disruption of homeostasis, which can integrate their signaling and co-activate in some conditions, thereby enhancing ER and mitochondrial function in response to stimuli that dysregulate proteostasis in these two organelles (Liu et al., 2014b; Lu et al., 2014b; Rainbolt et al., 2014). PERK is a putative signal-transducing crossroad connecting UPR^mt^ and UPR^ER^, which has been intensively explored. PERK is located at the ER-mitochondria contact sites, providing a PERK-mitochondria signaling pathway that enables PERK to detect stress derived from the proteostasis of both organelles. The PERK/ATF4 signaling axis, which is crucial for UPR^ER^ (Liu et al., 2014b), is also implicated in UPR^mt^ (Rainbolt et al., 2014). Activation of PERK/eIF2 signaling lowers the production of reactive oxygen species from the mitochondrial complex. Similar to ATF4, the ATF5 and CHOP transcripts also contain ORFs in their upstream untranslated region. Hence, ATF5 and CHOP levels would increase upon PERK activation by UPR^ER^ or UPR^mt^ (Harding et al., 2003). PERK is necessary for ATF5 expression, as genetic deletion of PERK prevents the increase in ATF5 levels in ER-stressed cells (Zhou et al., 2008; Teske et al., 2013). During ER stress, the levels of the UPR^mt^ transcripts, Lon protease, and mitochondrial HSP70 (GRP75) increased in PERK- eIF2- ATF4 (Lebeau et al., 2018). These findings imply that following ER stress, the PERK- eIF2- ATF4 pathway can promote collaboration between ATF4 and CHOP, consequently increasing the expression of ATF5 and enhancing the production of mitochondrial chaperones, facilitating the correct formation of proteins and proteases that degrade abnormal proteins (Figure 1).

Mitochondria are known as vital organelles that are involved in metabolism (especially energy metabolism), and maintaining homeostasis is not only important for the organelle itself, but also for the whole cell. As an essential mechanism to cope with homeostasis disruption, UPR^mt^ and UPR^ER^ participate in the development of various metabolic diseases, such as diabetes, obesity, and fatty liver (Qureshi et al., 2017; Yan et al., 2020). For instance, evidence has indicated that both UPR^mt^ and UPR^ER^ participates in obesity development. Obesity is a metabolic disease caused by the fact that fat cannot be consumed efficiently and is instead produced excessively and accumulates for a long time. Fat can be oxidized in mitochondria as a way of consumption, and activating UPR^mt^ facilitates efficient fat utilization, therefore UPR^mt^ alleviates obesity (Bhaskaran et al., 2017; Chung et al., 2017). The genes of HSP60, SIRT1, SIRT3, LONP1 and CLPP related to UPR^mt^ were significantly downregulated in the individuals with larger body mass index. Furthermore, the activation of UPR^mt^ efficiently facilitates fat utilization in an unsaturated fat diet, whereas inhibiting UPR^mt^ promotes the accumulation of fat in a saturated fat diet. In addition, UPR^mt^ promotes systemic energy expenditure via GDF15 that is capable of enhancing lipid consumption in muscle and adipose tissues, which in turn contributes to protecting the body from obesity. To summarize, in terms of stress, UPR^mt^ mainly regulates metabolic alterations through augmenting glycolysis and restraining OXPHOS (Jukarainen et al., 2016; Bhaskaran et al., 2017; Chung et al., 2017). In addition, the expression of ER stress markers are enhanced in obesity models and ER stress is related to the development of leptin resistance, which is an obesity inducer. The leptin hormone increases energy expenditure, which plays a protective role against obesity. Leptin-induced STAT3 phosphorylation can be inhibited by ER stress inducers, which are associated with leptin-resistance and body weight gain. XBPs and ATF6 overexpression associated with UPR^ER^ alleviate ER stress and promote the transduction of leptin signaling. Additionally, mice with a XBPs deletion in neurons consequently reduced ER folding capacity, and displayed an obesogenic phenotype upon high fat diet feeding accompanied with decreased energy expenditure and hyperphagia. Furthermore, PERK phosphorylation levels are significantly increased and the STAT3 phosphorylation induced by leptin was markedly blunted in such mice (Ozcan et al., 2004; Hosoi et al., 2008; Zhang et al., 2008; Ozcan et al., 2009; Won et al., 2009; Cakir et al., 2013; Schneeberger et al., 2013; Lee and Ozcan, 2014; Williams et al., 2014). Notably, UPR^mt^ coordinating with UPR^ER^ participates in the development of metabolic diseases. For example, oxidative stressors such as ROS in mitochondria, associated with the occurrence and development of metabolic diseases, can initiate UPR^ER^ before activating UPR^mt^. UPR^ER^ can touch off UPR^mt^ via a ATF4-dependent manner. Moreover, LONP1, as a vital protease in UPR^mt^, can also participate in UPR^ER^. Therefore, the coordination between UPR^mt^ and UPR^ER^ must be remarkable to manage stress and maintain cellular homeostasis. However, the detailed mechanism related to the crosstalk between UPR^mt^ and UPR^ER^ in metabolism are lacking, and requires additional studies (Iqbal and Hood, 2014; Yang et al., 2018; Waldherr et al., 2019; Jiang et al., 2020)

### Coordination of UPR^mt^ and UPR^ER^ in T2D

T2D is a global health concern. It is the leading cause of morbidity and mortality worldwide. T2D is characterized by insulin resistance in peripheral tissues (skeletal muscles, adipose tissues, etc.) and pancreatic beta cell dysfunction. Initially, factors such as obesity led to insulin resistance, forcing pancreatic beta cells to exert excessive normal action for more insulin to maintain normal glucose levels. However, prolonged overstimulation results in beta cell dysfunction and death, ultimately leading to hyperglycemia and gradually leading to diabetes. UPR^mt^ plays a crucial role in T2D, from insulin resistance to beta cell dysfunction. Increased glucose and fatty acid metabolism in cells in hyperglycemia and hyperlipidemia result in increased ROS production and protein aggregation (Fernandes et al., 2020; Kos, 2020). Consequently, UPR^mt^ is activated to increase glucose metabolism and decrease ROS generation and protein aggregation (Fernandes et al., 2020). UPR^mt^ regulates OXPHOS and glycolysis to increase glucose metabolism. Chronic hyperglycemia impairs the UPR^mt^ in people with diabetes. UPR^mt^ has been observed to be decreased in the brains of insulin-resistant mice, mice lacking insulin signaling, and people with T2D (Fernandes et al., 2020). Moreover, it has been demonstrated that insulin working in the brain promotes UPR^mt^ and attenuates diet-induced weight gain (Wardelmann et al., 2019).

UPR^ER^ coordinates with UPR^mt^ in T2D by activating the PERK pathway. Downstream PERK has been reported to be involved in T2D. The homeodomain transcription factor of pancreas/duodenum homeobox protein 1 (Pdx1) and human diabetes genes partially regulate beta cell survival via direct regulation of the activating transcription factor ATF5. Significantly, the absence of ATF5 decreased the survival rate under stressful conditions. ATF5 is parallel to ATF4 downstream under the control of eIF4E-binding protein 1 (4ebp1), a component of the mammalian target of rapamycin (mTOR) pathway that slows the translation of proteins, based on loss-of-function and chromatin occupancy tests. Consequently, the absence of ATF5 decreased the stress-induced inhibition of almost the whole translation, thereby increasing the vulnerability of cells to stress-induced death (Pakos-Zebrucka et al., 2016). CHOP depletion has been demonstrated as a therapeutic method for reducing dysregulated insulin secretion and fatty liver pathology in T2D (Wardelmann et al., 2019).

PERK, a crucial sensor for responses to ER stress, is located in the MAMs, which is composed of membrane compositions from mitochondria and ER, has a closely related impact on the ER–mitochondrial interaction and mitochondrial apoptosis mediated by ROS (Verfaillie et al., 2012; Verfaillie et al., 2013). For example knocking out PERK in murine embryonic fibroblasts (MEFs) causes a disturbance to the ER–mitochondria association, consequentially causing disruption to ER morphology, decreasing the transduction of ROS signaling from ER to mitochondria, reducing the flux of Ca2+ from ER to mitochondria, and bringing about decline in mitochondrial apoptosis induced by ROS (Liu et al., 2013). The leucine-rich repeat kinase 2 (LRRK2) enzyme suppresses PERK-induced phosphorylation of the E3 ubiquitin ligases, parkin. In LRRK2 KO MEFs, parkin is phosphorylated by PERK, and mitofusin-2 (Mfn2) (located at the MAMs) is ubiquitinated and degraded, which causes the ER–mitochondria association to decrease (Toyofuku et al., 2020). Furthermore, PERK can form the PERK–Mfn2 complex and PERK–S1R complex contacting with Mfn2 and sigma-1 receptor (S1R), which are also involved in the composition of MAMs and sustain the stabilization of ER-mitochondrial contacts (Liu et al., 2013; Cao et al., 2021).

Disruption of Ca2+ homeostasis is to some extent related to metabolic disorders such as insulin resistance, obesity, and T2D in both humans and animals. For example, primary adipocytes isolated from humans with obesity and insulin resistance (Draznin et al., 1988) or obtained from rats with diabetes (Draznin et al., 1989) exhibited higher Ca2+ levels within the cell compared to ordinary individuals. Simultaneously, MAMs are the contact interfaces linking the outer membrane of mitochondria and ER membrane where the molecules between two organelles communicating with each other, also has a definition of structural membranes between the two compartments (Phillips and Voeltz, 2016). Notably, mitochondria and ER, shown as the two largest compartments storing Ca2+ in human cells, have Ca2+ ion transportation and the efficient and precise communication between the two organelles, relies on such a special membrane structure. Therefore, MAMs shoulder the responsibility of dynamically and efficiently transmitting Ca2+ signals between the ER and the mitochondria in physiology and pathology. Owing to Ca2+ handling proteins that are enriched in the sites of MAMs, regulating intracellular Ca2+ homeostasis is dependent on the functional coupling at the contact sites of ER-mitochondria when the cells cope with various pathophysiological and environmental stimuli and go through metabolic reprogramming (Theurey and Rieusset, 2017). In addition, there are abundant functional enzymes and proteins that participate in the metabolism of fatty acids and glucose locating at MAMs, thus MAMs are referred as an integrator of energy metabolism (Sala-Vila et al., 2016; Rieusset, 2017). In conclusion, we believe that MAMs has an intimate relationship with the occurrence of T2D.

**FIGURE 2:**
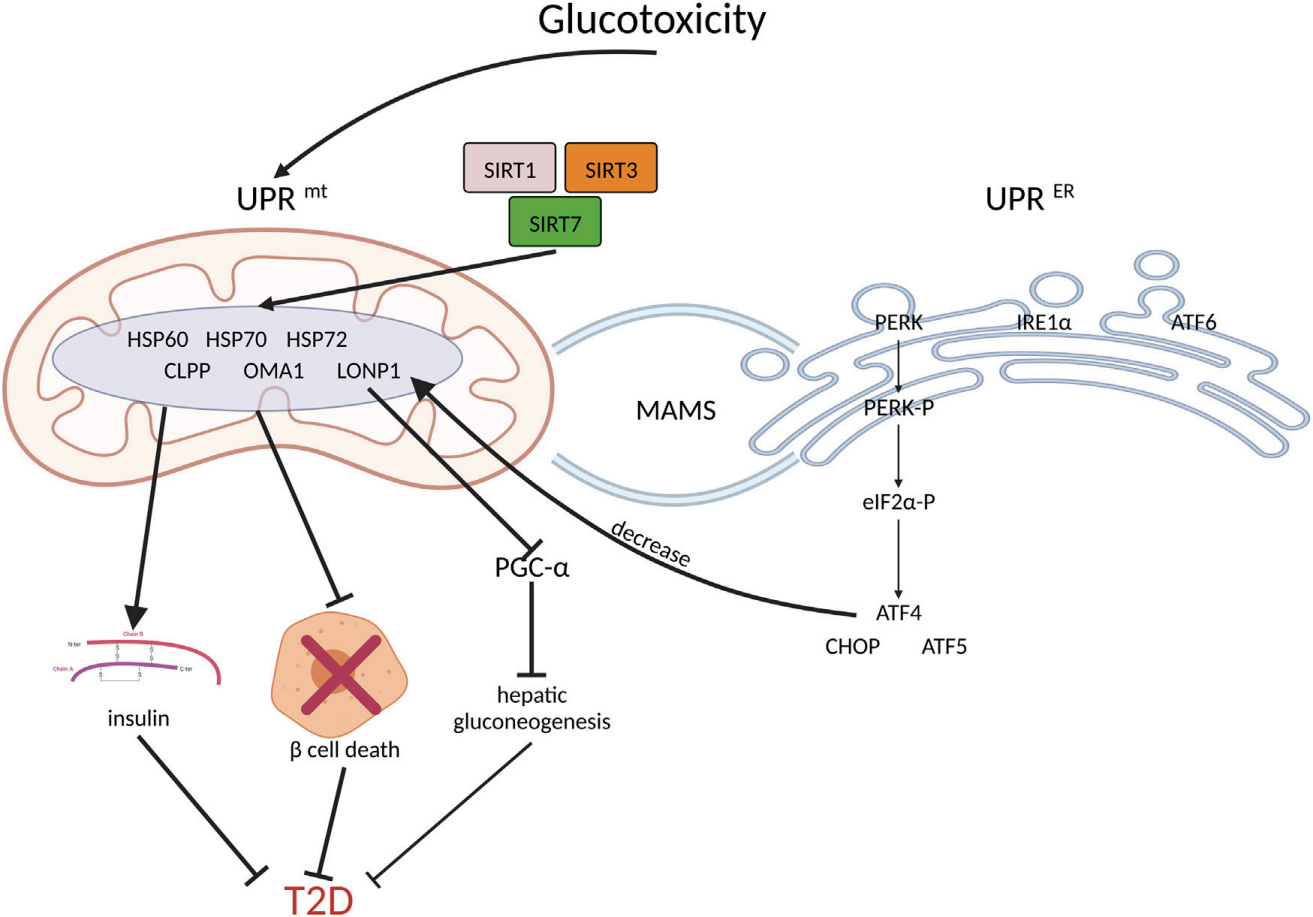
UPR^mt^ and coordinated UPR^ER^ increase insulin sensitivity, inhibit beta cell death and benefit T2D. Firstly, hyperglycemia induces UPR^mt^ and UPR^ER^. As the downstream of PERK, ATF4, CHOP and ATF5 will inhibit beta cell death; thus, they have beneficial effects on T2D management. Secondly, SIRT1, SIRT3 and SIRT5 activate UPR^mt^, which improves insulin sensitivity. In addition, the increased expression of mitochondrial chaperone proteins and proteases, such as HSP60, HSP70, HSP72, LONP1, CLPP, and OMA1, may reduce insulin resistance. Moreover, by inhibiting PGC-1a, LONP1 suppresses hepatic gluconeogenesis, thereby ameliorating hepatic insulin resistance.

In addition, other UPR^mt^ components, such as SIRT1, SIRT3, and SIRT5, have positive regulatory effects on insulin sensitivity (Owusu-Ansah et al., 2013; Jukarainen et al., 2016; Yong et al., 2021). HSP60, HSP72, and HSP70, which are mitochondrial chaperone proteins, substantially impact T2D (Lee et al., 2011; Kim et al., 2013; Kleinridders et al., 2013; Henstridge et al., 2014; Mottis et al., 2014; Lee et al., 2015; Aluksanasuwan et al., 2017). Moreover, high levels of mitochondrial proteases, including LONP1, CLPP, and OMA1, are also involved in protective action in T2D (Lee et al., 2011; Lee et al., 2015; Wu et al., 2019; Kalvala et al., 2020). (Figure 2).

A germline deletion of the CHOP gene, which encodes the C/EBP homologous protein as a transcription factor bound to enhancer, can prevent β cell failure in diabetes models (Song et al., 2008; Satoh et al., 2011; Maris et al., 2012). However, whether the CHOP deletion gives protection to β cells by the means of a cell-autonomous manner, remains unclear (Kempf et al., 2012).

Currently, researchers investigated whether there are therapeutic benefits through targeting CHOP specifically in β cells of mice. First, CHOP deletions in ß cells alleviated ER stress and the glucose-stimulated insulin secretion is delayed in the mice that are fed a high fat diet (HFD-fed mice). Second, specific CHOP deletion in ß cell can protect aged HFD-fed mice against liver hepatomegaly and steatosis and not influence basal glucose homeostasis. Third, the Ca2+ buffering capacity within ER is reduced and glucose-induced Ca2+ oscillations is regulated in terms of CHOP depletion in cells, causing a result of changes in transcriptional profile of the ER chaperone. Finally, there is decline of pancreatic insulin content and liver triglycerides under the operation of exerting CHOP antisense oligonucleotide conjugated with GLP1. In conclusion, these results suggest a novel therapeutic strategy via targeting CHOP in β cells to mitigate the development of T2D, while regulating insulin secretion and alleviating syndromes of consequent fatty liver disease (Yong et al., 2021).

As a member of TGF-β superfamily, the growth differentiation factor-15 (GDF-15) cytokine has an impact on the development of several metabolic disorders via plasmatic level regulation, including T2D (Kempf et al., 2012; Bao et al., 2019), cardiovascular diseases (Brown et al., 2002), obesity (Dostálová et al., 2009; Vila et al., 2011), non-alcoholic steatohepatitis (NASH) (Kempf et al., 2012), and various cancers (Brown et al., 2003; Koopmann et al., 2004). Moreover, there is some connection between the CHOP and GDF-15. The transcription factor CHOP, which is directly bound to the promoter region of GDF15, modulates the expression of GDF15. In addition, saturated fatty acids (SFAs) can significantly induce the expression and secretion of GDF15 (L'Homme et al., 2020). Therefore, GDF-15 may have a relationship with the development of several metabolic disorders, which could be a potential target of T2D.

## Conclusion and future perspectives

The study of UPR^mt^ is still in its infancy. Despite significant progress in the past few years, the crucial action of UPR^mt^ in metabolism, such as T2D, is gradually being understood. Here, we describe our current understanding of UPR^mt^ and its crosstalk with UPR^ER^, mainly through the PERK signaling pathway. Furthermore, we discuss the functions and molecular mechanisms of UPR^mt^ and coordinated UPR^ER^ in the context of T2D. Although our knowledge of the topics in this review has largely improved, many fundamental questions remain unanswered: Do UPR^ER^ components other than PERK also affect UPR^mt^? Does UPR^mt^ also regulate UPR^ER^? Which components of UPR^mt^ respond to insulin resistance development in T2D? Answers to these questions will help identify new candidate interventions for T2D.
